# Pharmacological Activity of Cha-Miang (*Camellia sinensis* var. *assamica*) in High Fat Diet-Induced Insulin-Resistant Rats

**DOI:** 10.3390/life14111515

**Published:** 2024-11-20

**Authors:** Jiraporn Laoung-on, Artorn Anuduang, Chalermpong Saenjum, Somdet Srichairatanakool, Kongsak Boonyapranai, Sakaewan Ounjaijean

**Affiliations:** 1 Office of Research Administration, Chiang Mai University, Chiang Mai 50200, Thailand; jiraporn.l@cmu.ac.th; 2 Research Institute for Health Sciences, Chiang Mai University, Chiang Mai 50200, Thailand; a.anuduang@gmail.com (A.A.); kongsak.b@cmu.ac.th (K.B.); 3 Department of Pharmaceutical Sciences, Faculty of Pharmacy, Chiang Mai University, Chiang Mai 50200, Thailand; chalermpong.s@cmu.ac.th; 4 Department of Biochemistry, Faculty of Medicine, Chiang Mai University, Chiang Mai 50200, Thailand; somdet.s@cmu.ac.th

**Keywords:** anti-obesity, high-fat-diet, liver biomarker, *Camellia sinensis*, antioxidant, lipid accumulation

## Abstract

Obesity, a major global health concern, is represented by an accumulation of adipose tissue and body mass, leading to a higher incidence of non-communicable diseases (NCDs). *Camellia sinensis* var. *assamica*, known as cha-miang, contains catechin and its derivatives, which have been reported to affect several health-related concerns such as anti-cancer, anti-inflammatory, anti-hyperlipidemia, and against cardiovascular disease. The research aimed to examine the anti-obesity and insulin resistance effects of fresh cha-miang extract (CME) and fermented cha-miang extract (FCME), and to investigate the anti-obesity and anti-diabetic effects of CME and FCME in obese rats generated by a high-fat diet. The extracts demonstrated significant antioxidant potential, with CME demonstrating greater DPPH radical scavenging activity, whereas FCME excelled in ABTS radical scavenging. In the animal model, CME and FCME significantly reduced body weight, plasma insulin levels, insulin resistance, and accumulation of fat compared to the obese control group. Moreover, plasma biochemical analysis indicated that both extracts enhanced lipid profiles by reducing cholesterol, triglycerides, and LDL-cholesterol levels, while elevating HDL-cholesterol. Histological analysis revealed decreased hepatic fat accumulation, especially when extracts were concurrently treated with metformin. The research indicates that CME and FCME, especially in conjunction with metformin, have potential anti-obesity and anti-insulin resistance benefits attributed to their abundant polyphenolic content and antioxidant characteristics. This indicates that cha-miang may serve as an effective option for the management of obesity and metabolic diseases.

## 1. Introduction

Obesity, a significant worldwide health issue, is defined by an increase in adipose tissue and body weight, resulting in more non-communicable diseases (NCDs) [1]. Metabolic syndrome, which is associated with an elevated risk of several chronic illnesses such as cancer, fatty liver, high blood pressure, hyperlipidemia, atherosclerosis, and obesity, is mostly caused by obesity [2,3]. Obesity results from an ongoing imbalance between caloric consumption and energy expenditure, leading to the accumulation of excess calories as fat in adipose tissue [4]. Moreover, obesity-induced visceral fat accommodation leads to induced liver lipoprotein production that affects triglyceride-rich VLDL levels, low-density lipoprotein (LDL) and total cholesterol levels and results in increased necrosis of blood vessels and induced insulin resistance [5]. Because obesity reduces life expectancy by lowering quality of life, reducing obesity may also reduce mortality, morbidity, and obesity-related disorders [5]. Although limiting calories and physical activity can successfully reduce obesity, anti-obesity medications may also be employed to regulate it by decreasing appetite and inhibiting fat absorption [6]. A multitude of innovative synthetic compounds are under investigation, concurrently focusing on the exploration of natural materials as alternative medicines [7,8]. A multitude of herbal therapies are currently accessible for rapid weight reduction through the stimulation of satiety and the acceleration of metabolism, serving effectively as anti-obesity agents [7].

Different kinds of tea leaves (*Camellia sinensis*) are extensively consumed worldwide as tea infusions [9]. In Thailand, Assam tea (*Camellia sinensis* var. *assamica*), known as cha-miang, is a small shrub that belongs to the Theaceae family [10]. The previous reported that tea leaves contain several biological properties of phenolic compounds, including antioxidant activities, antimutagenic effects, anticarcinogenic effects, prevention of nitrosation, and suppression of tumor [10]. Assam tea is often traditionally manufactured as fermented miang and has been widely consumed since ancestral times [11]. There is a great deal of regional variation in the traditional methods used for producing miang [11]. After collecting the leaves into a small bundle, they are steamed and left to ferment naturally in a bamboo basket for a period of several days to a year [10]. Chemical element analysis demonstrated that catechins represent a significant amount of the polyphenol content in fresh tea leaves, accounting for 60% to 70% of the total [12]. According to the research, the miang production process yields mostly flavonoids and polyphenols in its byproducts, which include fermented goods, wastewater, and fresh leaves [13]. Similar to other types of tea, these polyphenols include catechin and catechin derivatives, but the concentrations of the active components in each variety of tea differ significantly [10].

Catechins have a variety of beneficial pharmacological effects, including those against cancer, high blood pressure, inflammation, diabetes, obesity, hyperlipidemia, hypocholesterolemia, and cardiovascular disease [14,15,16]. In addition, the research group has also found that catechins in green tea can bind iron, helping to slow down iron overload and resist excessive free radicals in mice [17]. Moreover, previous reports indicated that fresh cha-miang and fermented cha-miang contain gallic acid, epigallocatechin, caffeine, catechin, epicatechin, and gallocatechin gallate, and they presented no harm to rats in acute and sub-acute toxicity studies [18]. However, the pharmacological activity of fresh cha-miang and fermented cha-miang on obesity and insulin resistance has insufficient scientific reports. Therefore, this study aims to investigate the antioxidant activity and its effect on high-fat diet-induced insulin resistance rats.

## 2. Materials and Methods

### 2.1. Sample Preparation and Extraction

In this study, cha-miang (*Camellia sinensis* var. *assamica*) leaf extracts were prepared from fresh leaves and fermented leaves (FCME). The fresh and fermented leaf samples were collected in September 2016 and originated from Pang Ma-O village, Chiang Dao District, Chiang Mai, Thailand. The specimens were transferred from the research project of Integrated Research of Miang (*Camellia sinensis* var. *assamica*) for Local Wisdom Inheritance and Economic Value, and were identified and authenticated at Herbarium, Faculty of Pharmacy, Chiang Mai University [10,19,20,21]. However, the transferred specimens were deposited at the Research Institute for Health Sciences, Chiang Mai University (00159). The fermented leaves were prepared following the previous study [21]. The samples were dried at 100 °C for 48 h, homogenized and extracted with hot distilled water at 100 °C for 1 h with 120 rpm of shaking (Fresh cha-miang leave extract: CME and fermented cha-miang leave extract: FCME), and the extract was dried by spay dryer with 12.56% yield of CME and 14.32% yield of FCME. The dried extracts were stored at –20 °C before experimentation.

### 2.2. Antioxidant Properties Assay

The free radical scavenging activity of the CME and FCME was determined using ABTS [22] and DPPH radical scavenging assay [23]. After the experiment procedure, the absorbance of ABTS and DPPH was measured using a microplate reader (BMG LABTECH, Offenburg, Germany) at 734 and 515 nm, respectively. Trolox and Butylated hydroxytoluene (BHT) were used as a standard.

### 2.3. Animals

Wistar rats (200–250 g, 6–8 weeks old) were obtained from the animal house of the National Laboratory Animal Center at Mahidol University (Nakhon Pathom, Thailand). Two or three rats were housed in each cage under standard environmental conditions (25 ± 2 °C temperature and 12-h light/dark cycle) and were maintained on a commercially balanced diet and water. All experimental procedures were approved by the Animal Ethics Committee, Faculty of Medicine, Chiang Mai University (38/2559) and agreed with the institutional guides for the Animal Care and Use of Laboratory Animals.

### 2.4. Experimental Design

A total of seventy-eight adult male Wistar rats were provided with feed and filtered water. Forty-eight male rats were induced to be insulin-resistant by a high-fat diet (HFD) for 12 weeks [24]. After acclimatization and inducing, all rats were divided into 13 groups (*n* = 6 per group). Group I–V were administered a normal control diet (NCD), whereas group VI-XIII were randomized to a high-fat diet (HFD).

- Group I (control: N-C): rats in this group were administered orally 1 mL/day of distilled water.
- Group II and III (CME): rats were administered orally CME at doses 150 (N-LCME) and 300 (N-HCME) mg/kg BW.
- Group IV and V (FCME): rats were administered orally FCME at doses 150 (N-LFCME) and 300 (N-HFCME) mg/kg BW.
- Group VI (obese: O-C): rats in this group were administered orally 1 mL/day of distilled water.
- Group VII (positive control): rats were administered orally Metformin at the doses 5 (O-Met) mg/kg BW.
- Group VIII and IX (obese and CME): rats were administered orally CME at doses 150 (O-LCME) and 300 (O-HCME) mg/kg BW.
- Group X and XI (obese and FCME): rats were administered orally FCME at doses 150 (O-LFCME) and 300 (O-HFCME) mg/kg BW.
- Group XII (Metformin and CME: O-Met+CME): rats were administered orally metformin 5 mg/kg BW and CME 150 mg/kg BW.
- Group XIII (Metformin and FCME: O-Met+FCME): rats were administered orally metformin 5 mg/kg BW and FCME 150 mg/kg BW.

The dose of CME and FCME in this study was correlated with the safety dose in the subacute toxicity study, which is 300 mg/kg [18]. All groups were orally treated by gavage needle daily for 12 weeks.

### 2.5. Measurement of Body Weight and Organ Weight

Body weights were recorded before initiating the treatments and also after each week of the interventions. All rats were anesthetized after 12 weeks of experimentation. Then, livers, kidneys, spleen, heart, and pancreas were removed, trimmed, and weighed before histological evaluation.

### 2.6. Insulin Resistant Assessment

Blood was collected from the tail vein after fasting for 8–12 h. Fasting blood glucose was measured using a portable blood glucose meter (Accu Chek Performa, Roche, IN, USA). Moreover, blood samples were collected in heparinized blood collection tubes and centrifuged at 3000 rpm for 15 min. The serum was separated for insulin quantification using the Rat Ins1/Insulin ELISA Kit (Sigma, Burlington, NJ, USA). The homeostasis model assessment of insulin resistance (HOMA-IR) was calculated using the following equation [25].

### 2.7. Biochemical Analysis

The plasma cholesterol, plasma triglyceride, and LDL-cholesterol in plasma were determined with the Triglyceride Quantification Kit and Cholesterol Assay Kit (Abcam, Cambridge, UK). Alanine aminotransferase (ALT) and aspartate aminotransferase (AST) were measured using an automatic analyzer (Randox, West Virginia, CA, USA) [26].

### 2.8. Plasma and Liver Lipid Peroxidation Assay

Lipid peroxidation (LPO) was determined using the thiobarbituric acid-reactive substances (TBAR) method, which is the reaction between thiobarbituric acid (TBA) and malondialdehyde (MDA). In addition, the antioxidant level was measured by the superoxide dismutase (SOD) enzyme in plasma, which used an automatic analyzer (Randox, West Virginia, CA, USA) [27].

### 2.9. Histological Evaluation

After being sacrificed, the livers, kidneys, spleen, pancreas and hearts were rapidly isolated and fixed in a 10% formaldehyde solution. The tissues were processed using the paraffin procedure. This involved dehydrating them with increasing intensities of ethanol, cleaning the tissues with xylene, embedding them in paraffin wax, sectioning at a thickness of 3 µm using a rotary microtome, and staining with hematoxylin and eosin (H&E). The morphological features of each organ were examined using a light microscope (Olympus CX31, Olympus Corporation, Tokyo, Japan) at 400×.

### 2.10. Statistical Analysis

The data were presented as descriptive statistics, mean ± standard deviation (SD). The Kolmogorov–Smirnov test was applied to assess the normal distribution. Mean values of acute and sub-acute toxicity were determined by one-way ANOVA, followed by Duncan’s multiple-range test for normality. The mean values of the other parameters were analyzed using the Kruskal–Wallis test, followed by Mann–Whitney U tests to evaluate the between groups differences. All statistical analyses were performed using SPSS 22 statistical software (Chicago, IL, USA). A *p*-value < 0.05 was considered statistically significant.

## 3. Results

### 3.1. Antioxidant Properties

The antioxidant properties of the CME and the FCME are presented in Table 1. The ABTS radical scavenging of the CME and the FCME presented 18.14 ± 0.03 and 21.65 ± 0.02 mg Trolox equivalent/g plant extract. Additionally, the DPPH radical scavenging of the CME and the FCME presented 36.91 ± 0.01 and 11.53 ± 0.01 mg Trolox equivalent/g plant extract.

### 3.2. Effect of CME and FCME on Body Weight and Relative Organ Weight

The mean value of body weight and relative organ weight are presented in Table 2. The O-C and O-Met groups significantly increased their body weights compared to the normal control (N-C). In contrast, other groups were significantly decreased from the obese control, except O-LCME, showing no significant difference from the N-C and O-C groups. Moreover, the O-C group significantly decreased the relative kidneys and heart weight when compared to the control. However, there were no significant alterations in the relative livers, spleen, and pancreas weight when compared with the N-C and O-C groups.

### 3.3. Effect of CME and FCME on Plasma Glucose, Plasma Insulin, and Insulin Resistance

**Figure 1:**
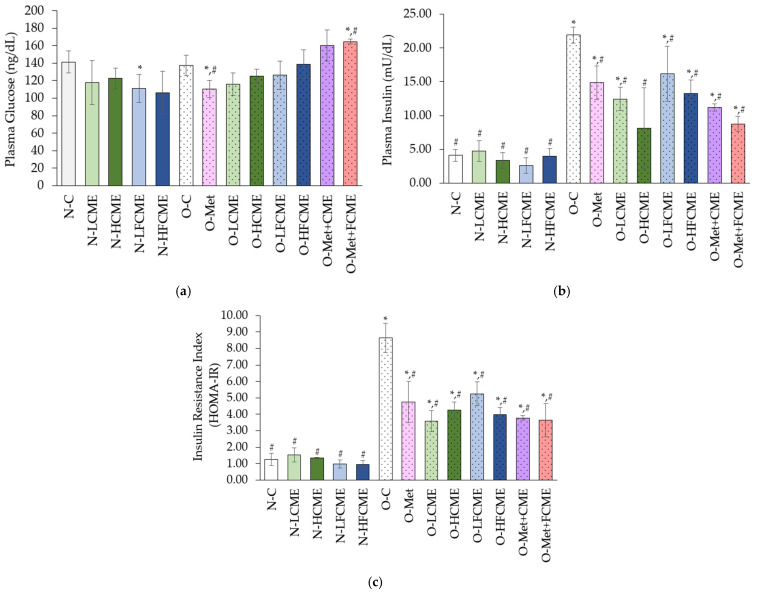
The mean value ± standard deviation (error bars) of plasma glucose (**a**); plasma insulin (**b**), and insulin resistance index (**c**). Data were obtained from six replications (*n* = 6). * Denotes the significant differences from the normal control group at *p* ≤ 0.05. # Denotes the significant differences from the obese control group at *p* ≤ 0.05.

The mean values of plasma glucose levels of the O-Met group decreased from the N-C and O-C groups, whereas the O-Met+FCME group was increased from the N-C and O-C groups. However, plasma insulin levels significantly increased in the O-C rats compared to the N-C group. All treatments in obese rats significantly declined the plasma insulin levels from the O-C group but were higher than the N-C rats except O-HCME, similar to the N-C group. Moreover, the O-C rats significantly increased insulin resistance index when compared to the N-C group, while all treatments of obese rats significantly declined insulin resistance index from the O-C group, albeit higher than the N-C group (Figure 1).

### 3.4. Effect of CME and FCME on Plasma Biochemistry

Plasma biochemistry includes cholesterol, triglyceride, LDL-cholesterol, HDL-cholesterol, ALT, and AST levels, as demonstrated in Table 3. The O-C group significantly increased cholesterol, triglyceride, LDL-cholesterol, ALT, and AST levels compared to the N-C group. All groups significantly decreased cholesterol levels in the O-C rats, similar to the N-C group. Additionally, the triglycerides significantly decreased in all groups, with the exception of N-HCME, O-LCME, O-HCME, and O-LFCME. The O-Met+FCME group was significantly decreased from the O-C and N-C groups. All groups, except for O-Met, O-LCME, O-HCME, and O-LFCME, showed significantly lower LDL-cholesterol levels when compared to the O-C rats, similar to the N-C group. However, the N-HCME group significantly lowered the LDL cholesterol levels when compared to the O-C and N-C rats. Furthermore, all treatments of obese rats significantly reduced their ALT and AST levels compared to the O-C group, except for the O-LFCME group. However, the HDL cholesterol levels significantly increased in all groups compared to the O-C and N-C groups, with the exception of the N-LCME and N-HFCME groups.

### 3.5. Effect of CME and FCME on Plasma and Liver Lipid Peroxidation

**Figure 2:**
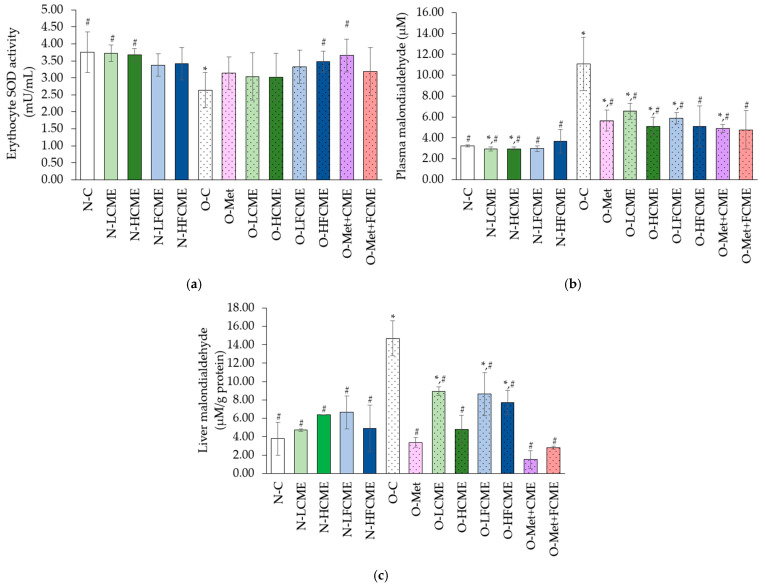
The mean value ± standard deviation (error bars) of erythocyte SOD (**a**); plasma malondialdehyde (**b**), and liver malondialdehyde (**c**). Data were obtained from six replications (*n* = 6). * Denotes the significant differences from the normal control group at *p* ≤ 0.05. # Denotes the significant differences from the obese control group at *p* ≤ 0.05.

Erythrocyte SOD activity significantly declined in the O-C group when compared to the N-C group, in which the O-HFCME and O-Met groups significantly increased this activity from the O-C group (Figure 2a). Plasma malondialdehyde (MDA) and liver MDA were significantly increased in the O-C rats when compared to the N-C group, in which all treatments of obese rats significantly decreased in the plasma MDA and liver MDA from the O-C group. The O-Met+FCME group showed decreases in plasma MDA, similar to the N-C group. Moreover, the O-Met, O-HCME, O-Met+CME, and O-Met+FCME groups decreased in liver MDA similarly to the N-C group (Figure 2b,c).

### 3.6. Effect of CME and FCME on Oragans Histological Features

**Figure 3:**
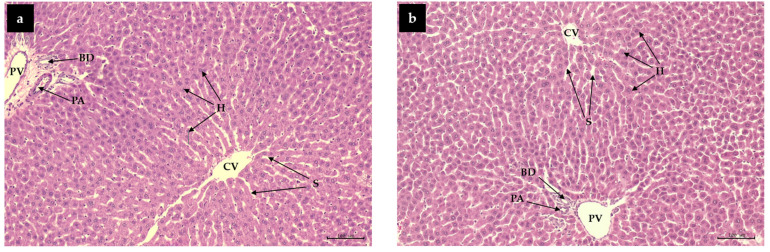

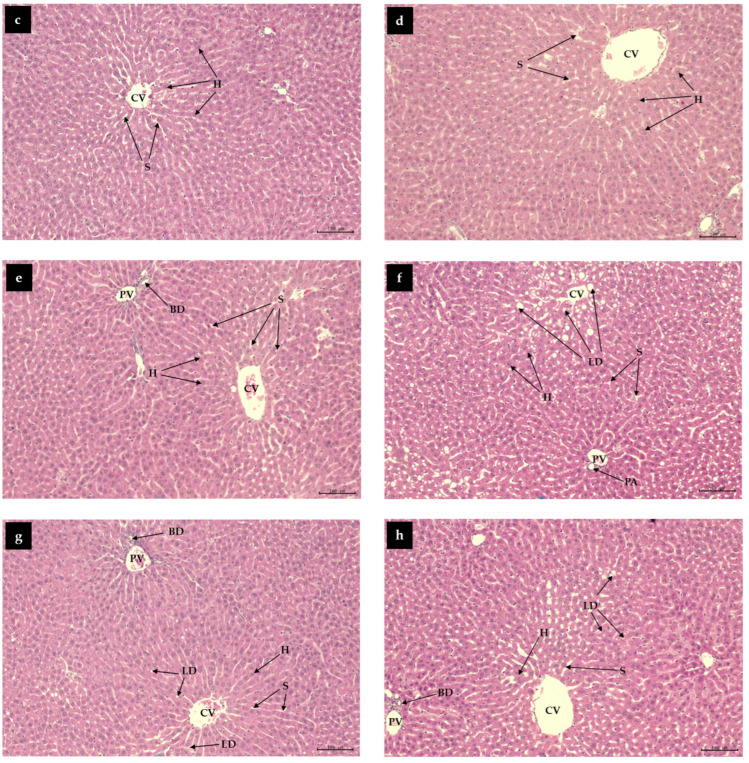

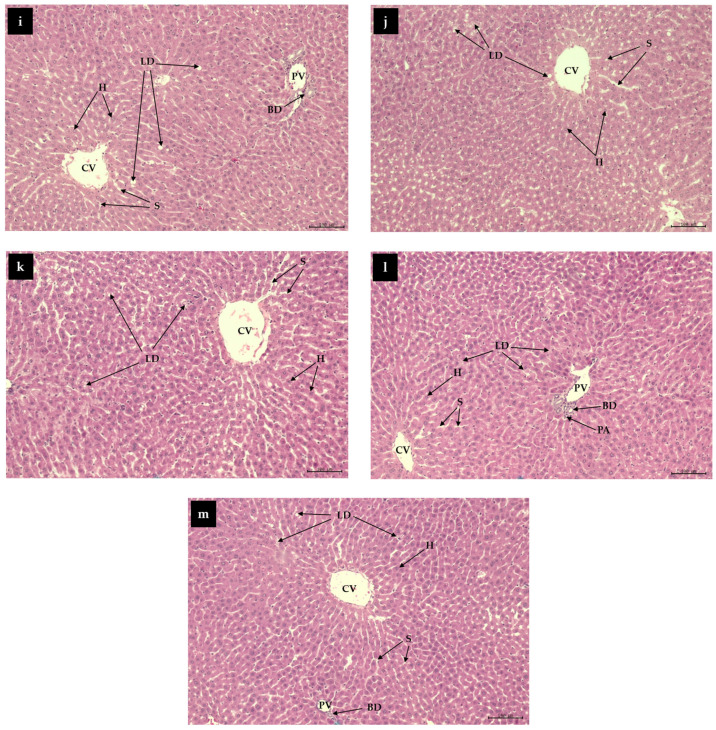
Photograph of histological feature of the liver using hematoxylin and eosin (H&E) staining and showed at a magnification of 100×. (**a**) normal control; (**b**) LCME; (**c**) HCME; (**d**) LFCME; (**e**) HFCME; (**f**) obese control; (**g**) O-Met; (**h**) O-LCME; (**i**) O-HCME; (**j**) O-LFCME; (**k**) O-HFCME; (**l**) O-Met+CME; and (**m**) O-Met+FCME. CV: central vein; H: hepatocytes; S: hepatic sinusoids; PV: portal vein; PA: portal artery; BD: bile duct; LD: lipid droplets.

The histological characteristics of essential organs were demonstrated (Figure 3). The tissues indicate that the high-fat diet increased fat accumulation in the liver (fatty liver), with macrovascular liver steatosis. The liver cells were expanded and flocculent, with changes in the cytoplasm and nucleus. Droplets of various sizes were found, indicating the development of non-alcoholic fatty liver disease (NAFLD) without any significant effects on other organs. The hepatic histological feature showed decreasing lipid droplets in the CME and FCME groups in the experimental rats that received the high-fat diet, especially co-administering CME and FCME with metformin (Figure 3).

## 4. Discussion

In the current study, rats were induced into metabolic homeostasis by administering a high-fat diet (HFD) and producing experimental obesity. Usually, abnormalities in metabolism result in raised plasma lipids, defined by increased total cholesterol (TC), triglycerides (TG), and low-density lipoprotein (LDL) cholesterol [28]. Furthermore, HFD has induced hyperglycemia in rats [29]. This study investigated blood glucose levels and lipid levels as indicators of hyperglycemia and hyperlipidemia. In the current study, HFD was administered for 12 weeks, resulting in obesity and dyslipidemia, as demonstrated by weight gain and elevated cholesterol values [24,30]. This study found that the HFD rats increased body weight gain from the normal control but decreased relative kidney weight and relative heart weight. Hepatic steatosis, enlarged adipocytes, hyperlipidemia, and disrupted blood glucose regulation are characteristic features of obesity caused by excessive fat deposition [31]. Numerous studies have established a significant correlation between a high-fat diet and oxidative stress in both humans and animals [32,33,34]. Obesity resulting from a high-fat diet has been associated with oxidative stress, which elevates the synthesis of oxidized LDL cholesterol, implicated in the development of dyslipidemia, atherosclerosis, and cardiovascular disease [30,35]. Currently, various natural phytochemical compounds obtained from fruits and vegetables exhibit inhibitory effects on obesity and obesity-related metabolic syndrome [36].

*Camellia sinensis*, commonly known as tea, is the most widely consumed beverage around the world and provides an essential source of natural antioxidants [9]. Cha-miang (*Camellia sinensis* var. *assamica*) is largely cultivated in Northern Thailand [11]. The report showed that 77% of fresh tea leaves produced in Thailand were processed into dried tea, whilst 23% were applied to produce miang, a traditional Lanna fermented tea leaf [11]. Fresh cha-miang and fermented cha-miang were extracted with hot distilled water, providing a high total phenolic content and showing cathine-delivering compounds including gallic acid, epigallocatechin, caffeine, catechin, epicatechin, and gallocatechin gallate [19]. The previous reported that the total phenolic content of fresh cha-miang (CME) and fermented cha-miang (FCME) was 2700.56 ± 12.73 and 1539.44 ± 30.71 mg gallic equivalent/g extract, respectively. Catechin and related-compound profiling of CME and FCME demonstrated gallic acid, gallocatechin, epigallocatechin, caffeine, catechin, epicatechin, epigallocatechin gallate, gallocatechin gallate, and epicatechin gallate and, at the dose of 300 mg/kg, no toxicity effect in rats [18]. Catechins are dietary polyphenolic compounds that are biologically active molecules attributed to plenty of beneficial effects on health [37]. The antioxidant activity of the CME and FCME was evaluated by the DPPH radical scavenging and ABTS radical scavenging. The CME showed high potential in DPPH radical scavenging, whereas the FCME showed high potential in ABTS radical scavenging. DPPH radical scavenging and ABTS radical scavenging are based on a single electron transfer mechanism [38]. The CME and FCME had a potential for antioxidant capacity by the electron transfer mechanism. The results indicated that the CME and FCME contained a high concentration of catechin derivatives as polyphenolic compounds, which demonstrate crucial antioxidant capacity.

After a 12-week treatment period, there was a significant anti-obesity effect of the CME and FCME, especially the co-administering metformin and CME at a dosage of 150 mg/kg/day in HFD-induced obese rats in comparison to the O-C rats. Rats administered CME and FCME demonstrated significant decreases in body weight, while enhancements in kidney and heart weights. The increase in overall body weight significantly impacts visceral organs, especially the liver via fat accumulation [39]. In this study, the O-C rats increased plasma insulin and insulin resistance, while obese rats with CME, FCME, and metformin showed a reduction of plasma insulin and insulin resistance similar to the normal control. Insulin is an essential hormone that regulates glucose levels. Various studies indicate a strong correlation between obesity and type 2 diabetes with insulin resistance [40]. Our research shows that CME and FCME may help regulate insulin resistance, which is the primary cause of type 2 diabetes. In a recent study, HDL-cholesterol levels were increased in CME, FCME, and metformin-supplemented obese rats when compared to the obese control group. Cholesterol, triglyceride, and LDL-cholesterol levels were increased in obese control rats, while CME, FCME, and metformin-supplemented obese rats presented reduced levels. An altered lipid profile is the primary indicator of liver illness, with elevated LDL and decreased HDL values serving as significant risk factors for coronary heart disease [35]. Cardiovascular diseases are associated with elevated cholesterol, triglycerides, and LDL-cholesterol levels in plasma [41]. The reduction in cholesterol, triglycerides, and LDL-cholesterol levels indicates that CME and FCME could reduce the risk of cardiovascular diseases.

The liver detoxifies and synthesizes protein utilizing a complex mix of enzymes, of which ALT and AST levels, liver biomarkers related to obesity, were investigated. Our investigation demonstrated higher ALP and AST in the O-C group. In contrast, CME and FCME-supplemented obese rats had nearly normal enzyme levels. The increased levels of ALT and AST suggest hepatic damage and nonalcoholic fatty liver disease, and correspond with type 2 diabetes [42]. The results indicated that the CME and FCME had the potential to reduce lever enzyme levels and prevent type 2 diabetes.

Additionally, the O-C group decreased erythrocyte SOD activity while increasing plasma and liver MDA when compared to the N-C group. However, the CME and FCME-supplemented obese rats had enhanced SOD activity and reduced plasma and liver MDA from the O-C group. These parameters are related to antioxidant and radical scavenging activity [43]. This is the same as the green tea extract’s decreased MDA levels and enhanced SOD activity [44]. The antioxidant efficacy of CME and FCME may be related to an increased number of total polyphenols present in the plant. Polyphenolic compounds may reduce xanthine oxidase activity, which is implicated in superoxide radical generation and the activation of antioxidant enzyme activity [45]. The results are consistent with the result in in vitro antioxidant activity.

The histological examination of the liver indicates an increase in lipid droplets in O-C rats, whereas a reduction is found in the CME and FCME-supplemented obese groups. Obesity resulting from a high-calorie diet induces abnormalities in the structural and cellular composition of adipose tissues. However, these alterations result in an increase in adipocyte size [46]. Alterations in lipid transport and lipoprotein secretion can lead to the expansion of normal lipid droplets in the liver [35]. The size of lipid droplets was significantly reduced in the obese rat groups treated with CME, FCME, and metformin. Our findings indicate that the CME and FCME are beneficial in regulating whole body fat and guarding visceral organs against hypolipidemia via several metabolic pathways.

The current investigation indicates that CME, FCME, and metformin, especially co-administering metformin and CME, exhibit more beneficially influenced various obesity-related parameters. The second metabolism of CME and FCME had anti-obesity action via the inhibition of adipogenesis and fat accumulation by modulating the reduction of adipogenesis-related factors, the enhancement of lipolysis-related factors and improved glucose tolerance and insulin activity, consequently reducing damage-associated liver enzymes. Consequently, CME and FCME have been identified as significantly advantageous in obesity, and our study has established a reasonable pharmacological foundation for its application in this condition.

## 5. Conclusions

In conclusion, our data indicate that CME and FCME improve rats from obesity and associated problems generated by a high-fat diet. CME and FCME are associated with a reduction in body weight compared to the O-C group. CME and FCME decrease plasma insulin levels, insulin resistance, and plasma biomarkers associated with obesity and lipid accumulation. They also control hepatic biomarkers, as well as the normal dimensions of hepatocytes and adipocytes. Based on these findings, it appears that CME and FCME could be promising candidates for preventing obesity caused by a high-fat diet. The restorative mechanisms linked to obesity may be ascribed to the antioxidant properties of CME and FCME polyphenols. Enhancing redox balance pathways is a highly effective therapy approach for obesity and its associated adverse effects.

## Figures and Tables

**Table 1:** The percentage inhibition of ABTS and DPPH of CME and FCME.

Parameter	CME	FCME
ABTS (mg Trolox equivalent/g plant extract)	18.14 ± 0.03	21.65 ± 0.02
DPPH (mg Trolox equivalent/g plant extract)	36.91 ± 0.01	11.53 ± 0.01

**Table 2:** Effect of CME and FCME on body weight and organ weight.

Groups	Body Weight (g)	Relative Organs Weight (g/100 g BW)				
		Livers	Kidneys	Spleen	Heart	Pancreas
N-C	563.33 ± 25.17 ^#^	1.90 ± 0.16	0.43 ± 0.03 ^#^	0.18 ± 0.02	0.27 ± 0.02 ^#^	0.29 ± 0.04
N-LCME	530.00 ± 14.14 ^#^	1.92 ± 0.31	0.44 ± 0.03 ^#^	0.17 ± 0.02	0.30 ± 0.01 ^#^	0.24 ± 0.08
N-HCME	595.00 ± 77.78 ^#^	1.99 ± 0.44	0.43 ± 0.06	0.17 ± 0.03	0.29 ± 0.04 ^#^	0.27 ± 0.12
N-LFCME	530.00 ± 39.16 ^#^	2.05 ± 0.09	0.42 ± 0.05	0.19 ± 0.03	0.30 ± 0.06	0.30 ± 0.09
N-HFCME	530.00 ± 34.64 ^#^	2.01 ± 0.16	0.43 ± 0.02 ^#^	0.20 ± 0.02	0.27 ± 0.03 ^#^	0.27 ± 0.04
O-C	728.00 ± 74.63 *	1.96 ± 0.49	0.34 ± 0.07 *	0.15 ± 0.03	0.22 ± 0.04 *	0.22 ± 0.02
O-Met	705.00 ± 12.91 *	2.13 ± 0.16	0.38 ± 0.03	0.16 ± 0.03	0.27 ± 0.02 ^#^	0.24 ± 0.02
O-LCME	615.00 ± 75.06	2.11 ± 0.17	0.41 ± 0.04	0.18 ± 0.03	0.27 ± 0.03 ^#^	0.27 ± 0.07
O-HCME	612.50 ± 58.52 ^#^	2.10 ± 0.32	0.43 ± 0.04 ^#^	0.16 ± 0.02	0.26 ± 0.02	0.32 ± 0.05
O-LFCME	630.00 ± 59.58 ^#^	2.34 ± 0.44	0.41 ± 0.04	0.19 ± 0.03	0.26 ± 0.03	0.26 ± 0.06
O-HFCME	587.50 ± 78.90 ^#^	2.31 ± 0.24	0.41 ± 0.04 ^#^	0.16 ± 0.02	0.25 ± 0.03	0.26 ± 0.06
O-Met+CME	585.00 ± 49.50 ^#^	2.23 ± 0.07	0.46 ± 0.02 ^#^	0.19 ± 0.01	0.30 ± 0.02 ^#^	0.31 ± 0.06
O-Met+FCME	592.50 ± 51.88 ^#^	2.07 ± 0.14	0.43 ± 0.03 ^#^	0.17 ± 0.02	0.27 ± 0.03	0.29 ± 0.04

Body weight and relative organ weight were analyzed by the Kruskal–Wallis test followed by the Mann–Whitney U test (*n* = 6). * Denotes the significant differences from the normal control group at *p* ≤ 0.05. ^#^ Denotes the significant differences from the obese control group at *p* ≤ 0.05.

**Table 3:** Effect of CME and FCME on plasma biochemistry.

Groups	Cholesterol (mg/dL)	Triglyceride (mg/dL)	LDL-Cholesterol (mg/dL)	HDL-Cholesterol (mg/dL)	ALT (U/L)	AST (U/L)
N-C	73.33 ± 6.81 ^#^	75.66 ± 5.51 ^#^	5.06 ± 0.61 ^#^	23.85 ± 4.42	30.60 ± 6.28 ^#^	104.36 ± 24.71 ^#^
N-LCME	72.00 ± 8.00 ^#^	90.25 ± 4.50 *^,#^	5.68 ± 0.81 ^#^	28.92 ± 5.12	34.66 ± 5.28 ^#^	107.97 ± 10.98 ^#^
N-HCME	71.33 ± 2.31 ^#^	85.50 ± 11.32 ^#^	3.89 ± 0.74 *^,#^	35.13 ± 3.49 *^,#^	27.81 ± 6.65 ^#^	108.73 ± 7.23 ^#^
N-LFCME	76.25 ± 6.44 ^#^	89.25 ± 9.11 ^#^	5.45 ± 1.08 ^#^	31.41 ± 1.37 *^,#^	32.95 ± 4.26 ^#^	126.25 ± 2.77
N-HFCME	72.25 ± 2.22 ^#^	103.00 ± 20.42 ^#^	4.61 ± 1.37 ^#^	33.42 ± 4.41	25.74 ± 4.37 ^#^	102.26 ± 8.38 ^#^
O-C	121.00 ± 20.52 *	151.20 ± 10.27 *	25.36 ± 3.73 *	26.76 ± 3.95	117.13 ± 30.32 *	206.18 ± 28.73 *
O-Met	83.75 ± 7.32 ^#^	81.00 ± 5.44 ^#^	10.20 ± 0.70 *^,#^	44.43 ± 6.90 *^,#^	34.38 ± 7.21 ^#^	125.96 ± 12.56 ^#^
O-LCME	86.00 ± 10.39 ^#^	110.47 ± 11.89 *^,#^	15.27 ± 1.66 *^,#^	42.13 ± 5.28 *^,#^	84.95 ± 22.81 *^,#^	136.94 ± 4.33 ^#^
O-HCME	76.67 ± 7.37 ^#^	95.33 ± 4.93 *^,#^	10.26 ± 0.70 *^,#^	35.20 ± 4.86 *^,#^	54.88 ± 6.70 *^,#^	102.82 ± 11.91 ^#^
O-LFCME	89.00 ± 9.85 ^#^	103.17 ± 4.88 *^,#^	11.91 ± 1.17 *^,#^	34.48 ± 1.25 *^,#^	83.86 ± 31.81 *	174.86 ± 11.30 *
O-HFCME	81.67 ± 5.86 ^#^	83.67 ± 18.18 ^#^	6.85 ± 1.33 ^#^	36.56 ± 4.86 *^,#^	60.24 ± 12.68 *^,#^	105.07 ± 20.47 ^#^
O-Met+CME	85.00 ± 12.77 ^#^	69.60 ± 8.05 ^#^	5.09 ± 1.30 ^#^	34.14 ± 6.66 *^,#^	42.64 ± 2.98 *^,#^	90.28 ± 20.23 ^#^
O-Met+FCME	74.75 ± 9.98 ^#^	60.50 ± 5.39 *^,#^	5.02 ± 0.59 ^#^	34.68 ± 3.11 *^,#^	34.90 ± 9.81 ^#^	90.58 ± 11.00 ^#^

Cholesterol, triglyceride, LDL-cholesterol, HDL-cholesterol, ALT, and AST levels were analyzed by the Kruskal–Wallis test followed by the Mann–Whitney U test (*n* = 6). * Denotes the significant differences from the normal control group at *p* ≤ 0.05. ^#^ Denotes the significant differences from the obese control group at *p* ≤ 0.05.

## Data Availability

The authors declare that the data supporting the findings of this study are available within the article.
